# Proteomic Signaling of Dual-Specificity Phosphatase 4 (DUSP4) in Alzheimer’s Disease

**DOI:** 10.3390/biom14010066

**Published:** 2024-01-03

**Authors:** Erming Wang, Allen L. Pan, Pritha Bagchi, Srikant Rangaraju, Nicholas T. Seyfried, Michelle E. Ehrlich, Stephen R. Salton, Bin Zhang

**Affiliations:** 1 Department of Genetics and Genomic Sciences, Icahn School of Medicine at Mount Sinai, One Gustave L. Levy Place, New York, NY 10029, USA; erming.wang@mssm.edu (E.W.);; 2 Mount Sinai Center for Transformative Disease Modeling, Icahn School of Medicine at Mount Sinai, One Gustave L. Levy Place, New York, NY 10029, USA; 3 Icahn Institute for Data Science and Genomic Technology, Icahn School of Medicine at Mount Sinai, One Gustave L. Levy Place, New York, NY 10029, USA; 4 Nash Family Department of Neuroscience, Icahn School of Medicine at Mount Sinai, 1425 Madison Avenue, New York, NY 10029, USA; 5 Department of Biochemistry, Emory Integrated Proteomics Core, Emory University School of Medicine, 1510 Clifton Rd NE, Atlanta, GA 30329, USA; 6 Department of Neurology, Emory University School of Medicine, 100 Woodruff Circle, Atlanta, GA 30322, USA; 7 Departments of Neurology and Pediatrics, Icahn School of Medicine at Mount Sinai, 1425 Madison Avenue, New York, NY 10029, USA; 8 Brookdale Department of Geriatrics and Palliative Medicine, Icahn School of Medicine at Mount Sinai, New York, NY 10029, USA

**Keywords:** DUSP4, 5xFAD, mouse model, proteomics, phosphoproteomics, MaxQuant, co-expression network, Alzheimer’s disease

## Abstract

DUSP4 is a member of the DUSP (dual-specificity phosphatase) subfamily that is selective to the mitogen-activated protein kinases (MAPK) and has been implicated in a range of biological processes and functions in Alzheimer’s disease (AD). In this study, we utilized the stereotactic delivery of adeno-associated virus (AAV)-DUSP4 to overexpress DUSP4 in the dorsal hippocampus of 5xFAD and wildtype (WT) mice, then used mass spectrometry (MS)-based proteomics along with the label-free quantification to profile the proteome and phosphoproteome in the hippocampus. We identified protein expression and phosphorylation patterns modulated in 5xFAD mice and examined the sex-specific impact of DUSP4 overexpression on the 5xFAD proteome/phosphoproteome. In 5xFAD mice, a substantial number of proteins were up- or down-regulated in both male and female mice in comparison to age and sex-matched WT mice, many of which are involved in AD-related biological processes, such as activated immune response or suppressed synaptic activities. Many proteins in pathways, such as immune response were found to be suppressed in response to DUSP4 overexpression in male 5xFAD mice. In contrast, such a shift was absent in female mice. For the phosphoproteome, we detected an array of phosphorylation sites regulated in 5xFAD compared to WT and modulated via DUSP4 overexpression in each sex. Interestingly, 5xFAD- and DUSP4-associated phosphorylation changes occurred in opposite directions. Strikingly, both the 5xFAD- and DUSP4-associated phosphorylation changes were found to be mostly in neurons and play key roles in neuronal processes and synaptic functions. Site-centric pathway analysis revealed that both the 5xFAD- and DUSP4-associated phosphorylation sites were enriched for a number of kinase sets in females but only a limited number of sets of kinases in male mice. Taken together, our results suggest that male and female 5xFAD mice responded to DUSP4 overexpression via shared and sex-specific molecular mechanisms, which might underly similar reductions in amyloid pathology in both sexes while learning deficits were reduced in only females with DUSP4 overexpression. Finally, we validated our findings with the sex-specific AD-associated proteomes in human cohorts and further developed DUSP4-centric proteomic network models and signaling maps for each sex.

## 1. Introduction

Alzheimer’s disease (AD) is the most prevalent neurodegenerative disease and the most extensively studied cause of dementia [1]. Pathologically, AD is characterized by and manifests with neurofibrillary tangles formed from the improperly processed phosphorylated tau proteins in the intracellular space and the accumulation of amyloid beta plaques in the intercellular space [2,3]. These abnormal aggregates are associated with oxidative stress and inflammation [4], resulting in microglial activation and neurodegeneration in the brain [5] and further causing the impairment or even loss of normal cognitive function and memory as age advances. Age, apolipoprotein E ɛ4 (APOE), and sex are the three greatest risk factors for AD [6]. In fact, sex is an important variable for AD patient stratification and personalized treatment [7], and a recent study of a large number of transcriptomes shows profound sex-specific changes and network remodeling in AD [8]. Mechanistically, the sex-specific differential response to AD might be caused by the sex-specific differential transcriptional response to AD pathology [9]. Despite extensive studies investigating the risk factors and neuropathogenesis of AD, the etiology and molecular and cellular mechanisms underlying AD are still largely unknown [2,3], and the majority of the experimental drugs tested for AD have failed without showing significant efficacy [10]. 

Proteomics analyses have been utilized to investigate mechanisms underlying neurodegenerative diseases because alterations in protein expression correlate better with phenotypes than changes in RNA expression [11]. Protein expression can be regulated at multiple levels, including transcriptional and epigenetic control over gene activity and post-transcriptional modulation of RNA splicing, stability, and transport [12]. Comparison of transcriptomic and proteomic profiling has revealed that ~40% of the variation in protein expression is likely caused and regulated by post-transcriptional and translational/post-translational mechanisms [13,14]. Post-translational modifications (PTMs) regulate protein trafficking, function, and degradation, and thus, aberrant PTMs of disease-relevant proteins would trigger abnormal alterations in pathological pathways, leading to disease progression [10]. Many studies of neurodegenerative diseases, including AD, have characterized PTMs of disease-relevant proteins such as tau [15] and TDP-43 [16]. Globally, MS-based proteomic analysis using both label-free [17] and tandem mass tag (TMT)-labeled [13,18] approaches plus various enrichment strategies has emerged as an important paradigm to survey changes in the PTMs of AD patients and healthy controls. The results from these comprehensive surveys [13,17,18] on PTMs provide valuable insight into the biochemical signaling pathways that drive AD pathogenesis and progression [15].

Dual-specificity phosphatases (DUSPs) are a protein phosphatase subfamily with selectivity towards mitogen-activated protein kinases (MAPKs) [19]. DUSP4, a member of this family, has been shown to dephosphorylate MAPKs, including ERK, JNK, and p38 kinases. In human epileptic brains, DUSP4 appears to function as a feedback inhibitor of pro-epileptogenic MAPK signaling [20]. Mechanistically, DUSP4 was demonstrated to be in the PRMT1-DUSP4-p38 axis to modulate cell differentiation [21]. DUSPs, including DUSP4, have become an important focus of research in neurodegenerative diseases because of their identified contributions to many important biological processes, including neuroprotection, differentiation, and inflammation [19]. In our recent study [22], we investigated the roles of DUSP4 and its downstream network in developing learning behavior impairment and neuropathology in the 5xFAD amyloidopathy mouse model. We found that overexpression of DUSP4 improves learning behavior only in female 5xFAD, whereas β-amyloid load is reduced in both male and female mice [22]. Transcriptomics profiling plus pathway enrichment analysis further supported the idea that DUSP4 may modulate the AD phenotype in a sex-specific manner [22].

In the present study, we sought to perform proteomics and phosphoproteomics analyses of the 5xFAD mice with and without DUSP4 overexpression to identify proteins and phosphorylation modulated via DUSP4. We further compared our DUSP4-modulated proteomes with AD-associated protein signatures and networks derived from large proteomic studies of human postmortem brains with AD to understand how DUSP4 may contribute to AD pathogenesis. Finally, we developed sex-specific, DUSP4-centric proteomic network models and signaling maps. 

## 2. Materials and Methods

### 2.1. Animal Studies

The 5xFAD transgenic mice were obtained from Jackson Labs (Bar Harbor, ME; JAX#34840) and were maintained on a mixed B6/SJL genetic background as described [23]. Male and female 5xFAD and wildtype (WT) at 4 months of age were stereotactically infused with 1.0 μL of the Adeno-Associated Virus (AAV)5-GFP or AAV5-DUSP4 (4 × 10^12^ vg/mL) into dorsal hippocampus (dHc) (AP = −2.0 mm, ML = ±1.5 mm, and DV = −2.0 mm relative to Bregma) at a rate of 0.2 μL per minute. AAV5-GFP (control) and AAV5-mouse DUSP4 (VectorBuilder Inc., Chicago, IL, USA; AAV-5′ITR-CAG-mDUSP4-WPRE-BGHpA-3′ITR) (AAV5 serotype/AAV2 genotype) were prepared using the Vector Core at the University of North Carolina at Chapel Hill. All mice (Supplementary Data S1) were housed under standard conditions (12 h light–dark cycle with ad libitum access to food and water). All experimental procedures were conducted in accordance with the NIH guidelines for animal research and were approved by the Institutional Animal Care and Use Committee (IACUC) at the Icahn School of Medicine at Mount Sinai (ISMMS) (IACUC ID: AR202300000080/IACUC-2015-0122, Approval date: 13 March 2023; IACUC ID: AR202300000293/LA10-00447, Approval date: 24 August 2023; OLAW approved Animal Welfare Assurance of Icahn School of Medicine at Mount Sinai is D16-00069 (A3111-01)).

A selection of 4 months of age for the DUSP4 overexpression in mice was based on the following evidence. It has been reported that the memory function of 5xFAD begins to deteriorate between 4 and 5 months [24]. In addition, the assessment of spatial working memory, by Y-maze and Morris water maze, of 5xFAD mice showed that the impairment begins between 4 and 6 months of age [25,26,27]. Amyloid deposition is already detectable in the 5xFAD cortex by 2 months [25], so to determine whether DUSP4 overexpression can reduce plaque burden, we chose a relatively early time point in disease progression. Therefore, we chose 4 months of age to assess the efficacy of DUSP4 overexpression to reduce or delay the progression of memory deficits and amyloid deposition.

### 2.2. Hippocampal Tissue Collection

One month after the stereotactic infusion, mice were sacrificed and perfused with 20 mL ice-cold phosphate-buffered saline (PBS). The whole hippocampal tissues were extracted from both hemispheres of the brain through gross dissection. Then, the tissues were rinsed with PBS prior to storing at −80 °C.

### 2.3. Western Blotting

STAT3, APP, and DUSP4 protein levels were analyzed using western blot as described [22]. Briefly, equal amounts of protein (20 µg) from each sample were resolved using electrophoresis in precast 4–12% Bis-Tris gels (Bio-Rad, Hercules, CA, USA) and transferred to a polyvinylidene difluoride (PVDF) membrane using the iBlot system (Invitrogen, Waltham, MA, USA). Membranes were then incubated in Odyssey blocking buffer (92760001, LI-COR, Lincoln, NE, USA) for 1 h at room temperature before incubation with the following primary antibodies in a mixture of blocking buffer (92760001, LI-COR, Lincoln, NE, USA) and 0.1% Tween 20 at 4 °C overnight: anti-DUSP4 (1:1000, ab216576, Abcam, Boston, MA, USA); anti-Aβ (1:1000, 803001, Biolegend, San Diego, CA, USA); or anti-actin (1:1000, MAB1501, Millipore Sigma, Burlington, MA, USA). On the second day, membranes were washed with 0.1% Tween 20 in a phosphate-buffered saline (PBS) solution and then incubated with a mixture of secondary antibodies: goat anti-rabbit 800CW (1:15,000, LI-COR, Lincoln, NE, USA) and goat anti-mouse 680LT (1:20,000, LI-COR, Lincoln, NE, USA) in an Odyssey blocking buffer with 0.01% sodium dodecyl sulfate (SDS) and 0.1% Tween 20 at room temperature for 1 h. Then, the membranes were washed with 0.1% Tween 20 in PBS, followed by PBS. The membranes were analyzed using an Odyssey infrared imaging system (LI-COR, Lincoln, NE, USA). Protein bands were quantified using Odyssey Imager analysis software (ImageStudio, version 5.2.5) and were normalized using actin as an internal loading control. 

### 2.4. Proteomics/Phosphoproteomics Sample Processing, Phosphopeptide Enrichment, LC-MS/MS, and MaxQuant Analysis 

Please see Supplementary information for details.

### 2.5. Sex-Specific Differentially Expressed Protein (DEP) Analysis in Mouse

We used the label-free quantification (LFQ) intensity as the abundance for individual protein groups, termed proteins, throughout the present study. We next removed proteins that are either potential contaminants, contain no protein (reverse), or were only identified by site. We only retained proteins that have expression in more than half of the samples. We then log2-transformed the protein expression and imputed the missing values via the function in the R package ‘impute’ [28] with the default parameters. Finally, we normalized the protein expression via median centering [29]. To identify DEPs, we performed a pair-wise comparison to detect significant changes in protein level between any two experimental mouse groups (Supplementary Data S2) in each mouse sex using the moderated *t*-test implemented in the limma package [30,31]. DEPs were determined to have a nominal *p*-value < 0.05 (see Section 4).

### 2.6. Sex-Specific Differentially Expressed Post-translational Modification (DEPTM) Analysis in Mice

We preprocessed the mass spectrometry (MS)-based phosphoproteome profiling using the R package PhosPiR, which removes MaxQuant-marked reverse sequences and potential contaminants and summarizes the intensities for each phosphosite entry, termed PTM site (see Supplementary information). The expression level (intensity) at each PTM site was obtained following quantile normalization and low-rank approximation imputation [32]. We removed any PTM site with no gene name or PTM position information. The expression was further log2-transformed for the downstream analysis. To identify DEPTMs, we performed a pair-wise comparison to detect significant changes in the PTM level between any two experimental mouse groups (Supplementary Data S3) across mouse sex using the moderated t-test implemented in the limma package [30,31]. DEPTMs were determined to have a nominal *p*-value < 0.05 (Section 4).

### 2.7. Gene Set Variation Analysis (GSVA) on PTM Site Enrichment Analysis

We applied the site-centric pathway analysis [33] on our PTMs via the algorithm described in the R package GSVA [34]. We examined the PTMs for enrichment over the mouse database of PTM site-specific phosphorylation signatures (PTMsigDB) [33]. We used the PTM expression matrix as the input to calculate the enrichment score for the sets of PTM sites in the mouse PTMsigDB in each sample across sex via GSVA [34]. We then performed differential analysis on the enrichment scores using the limma package [30,31], which was followed by multiple test adjustments using the Benjamini–Hochberg (BH) method.

### 2.8. Gene Ontology (GO) Enrichment Analysis and Plot Visualization

We used the R package clusterProfiler [35] to identify biological processes that are up- and down-regulated in each comparison. For DEP enrichment analysis, we used the function gseGO [35] since we wanted to capture both activated and suppressed enrichment, whereas we used the enrichGO [35] function in the enrichment analysis for the protein signatures derived from DEPTMs. The plots were generated using the Cytoscape (3.7.2) and the R packages ComplexHeatmap [36], ggplot2, ggpubr, EnhancedVolcano, and SuperExactTest [37]. The R version was 4.2.0. 

### 2.9. Sex-Specific DEP Analysis in Human Cohorts

We performed DEP analysis in AD vs. NL over the proteomics profile in two human AD cohorts using the postmortem tissue from two different brain regions: the parahippocampal gyrus (PHG) for the Mount Sinai Brain Bank (MSBB) [38] and the prefrontal cortex (PFC) for the Religious Orders Study and Memory and Rush Aging (ROSMAP) [39,40] cohort, respectively. The processing, normalization, and co-variable adjustment for human proteomics are as previously described [41]. In the present study, we stratified the subjects by sex and then identified the sex-specific DEPs in AD compared to NL (Supplementary Data S4). We used the multiple test adjusted *p*-value < 0.05 as the criteria to identify the differentially expressed proteins (DEP) in a human study.

### 2.10. Co-Expression Network Analysis

Gene co-expression networks on the proteomes in the human cohorts were identified using Multiscale Embedded GEne co-expression Network Analysis (MEGENA) as described in [41,42].

### 2.11. Construction of DUSP4-Centric Gene Co-Expression Networks

We constructed DUSP4-centric consensus gene co-expression networks from eight datasets from three cohorts, including the MSBB (four brain regions), ROSMAP (one brain region), and HBTRC (three brain regions) [43]. The genes significantly correlated with DUSP4 (FDR < 0.05) were identified in each dataset. From the significant correlations, a directional voting method was applied to calculate the frequency of negative or positive correlations between DUSP4 and each other gene. The DUSP4-centric network was thus defined as a function of frequency threshold *n* (=1, 2, …, 8) [43]. We then projected the DUSP4-associated DEPs in each sex onto the DUSP4-centric network, thus obtaining male or female-specific DUSP4-centric networks, respectively. 

### 2.12. Development of DUSP4-Centric Signaling Maps

Our recent publication [44,45] identified about 70 AD-related GO terms, including pathways related to Aβ, oxidative stress, tau NFT, and synaptic function. We used these gene sets to assess the relevance of a gene signature of interest with AD. Specifically, the connection between the gene signature of a target and an AD gene set is quantified using the enrichment score (−log10(FDR)), where FDR was determined using Fisher’s exact test and multiple testing corrections. We only keep the connections with an FDR < 0.05; thus, the higher the score, the more relevant a target to a pathway (i.e., GO term). All the significant connections constitute the target’s signaling map in AD.

## 3. Results

**Figure 1:**
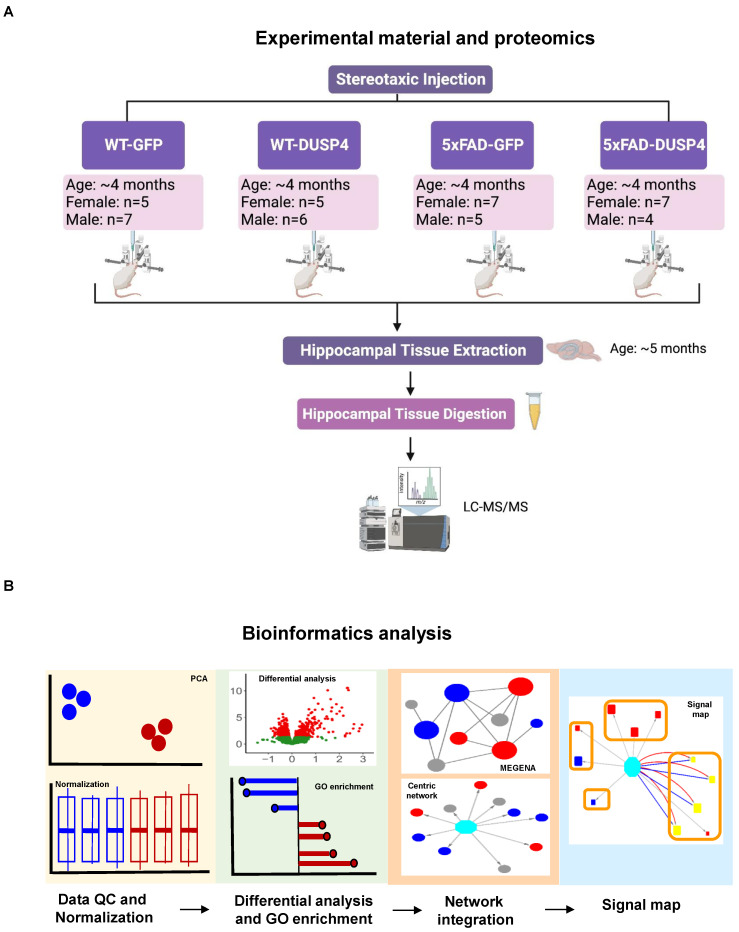
A schematic representation of the experimental materials, data collection, and bioinformatics workflow in this study. (**A**) Experimental materials and data collection, created with BioRender.com. The female and male 5xFAD and wildtype (WT) mice were injected with AAV5-DUSP4 or AAV5-GFP (control) into the hippocampus to over-express DUSP4 or the mock GFP at 4 months of age. The hippocampal tissues were then extracted from 5xFAD and WT over-expressing DUSP4 or the mock GFP one month after the surgery for subsequent LC-MS/MS analyses. (**B**) Downstream data processing and bioinformatics analysis workflow. The proteome and phosphoproteome data were first subject to quality control (QC) and normalization, followed by differential expression analysis to identify patterns of change (protein signatures or phospho-sites) in various comparisons, which were further used to query the gene ontology (GO) database for biological pathways and functional processes involved. Next, significant patterns of change (protein signatures or phospho-sites) in the mouse proteome and phosphoproteome were projected onto the human networks to investigate their relevance in human AD, and the construction of sex-specific gene-centric networks and signaling maps that reveal the biological relevance of the gene-centric networks. MEGENA, Multiscale Embedded Gene Co-expression Network Analysis; PCA, principal component analysis.

We performed both proteomic and phosphoproteomic analyses using the label-free quantification of MaxQuant [46,47,48] to analyze the mouse brain hippocampal samples extracted from four experimental groups that had been administered AAV-DUSP4 or AAV-GFP into dHc: 5xFAD-DUSP4 (*n* = 7 females, *n* = 4 males), 5xFAD-GFP (*n* = 7 females, *n* = 5 males), WT-GFP (*n* = 5 females, *n* = 7 males), and WT-DUSP4 (*n* = 5 females, *n* = 6 males) (Figure 1A, see Section 2 and Supplementary Data S1). As quality control (QC), we verified the genotypes of mice by the western blot analysis using the antibody (6E10) that specifically recognizes transgenic human amyloid precursor protein (APP) and using microscopic observation of the GFP protein activity/fluorescence (see Section 2). Based on this analysis, a male mouse originally identified as 5xFAD-GFP was re-classified as WT-GFP, and the downstream analysis was corrected. In addition, we conducted QC on the proteomic and phosphoproteomic data for further downstream processing (see Section 4). 

In the present study, we focused our analysis on the two most critical comparisons, i.e., 5xFAD-GFP vs. WT-GFP, to identify proteins and phosphoproteins that are regulated in the 5xFAD mouse model compared to WT, and in comparisons of 5xFAD-DUSP4 to 5xFAD-GFP, to investigate the impact of DUSP4 overexpression on the proteome/phosphoproteome in 5xFAD. To simplify the presentation, we termed the comparison 5xFAD-GFP vs. WT-GFP as 5xFADvsWT, and 5xFAD-DUSP4 vs. 5xFAD-GFP as 5xFAD-DUSP4vs5xFAD.

Furthermore, we used the nominal *p* < 0.05 as a cut-off to include the proteins/phosphoproteins regulated via DUSP4 overexpression. Our experimental validation of selected proteins and integration with human proteomics showed that this cut-off is an effective criterion for determining the proteomic/phosphoproteomic signatures regulated by DUSP4 (see Section 4). Figure 1B highlights the bioinformatics workflow for data analysis and integration. 

### 3.1. Substantial Numbers of Differentially Expressed Proteins (DEPs) Were Regulated in 5xFAD and via DUSP4 Overexpression

**Figure 2:**
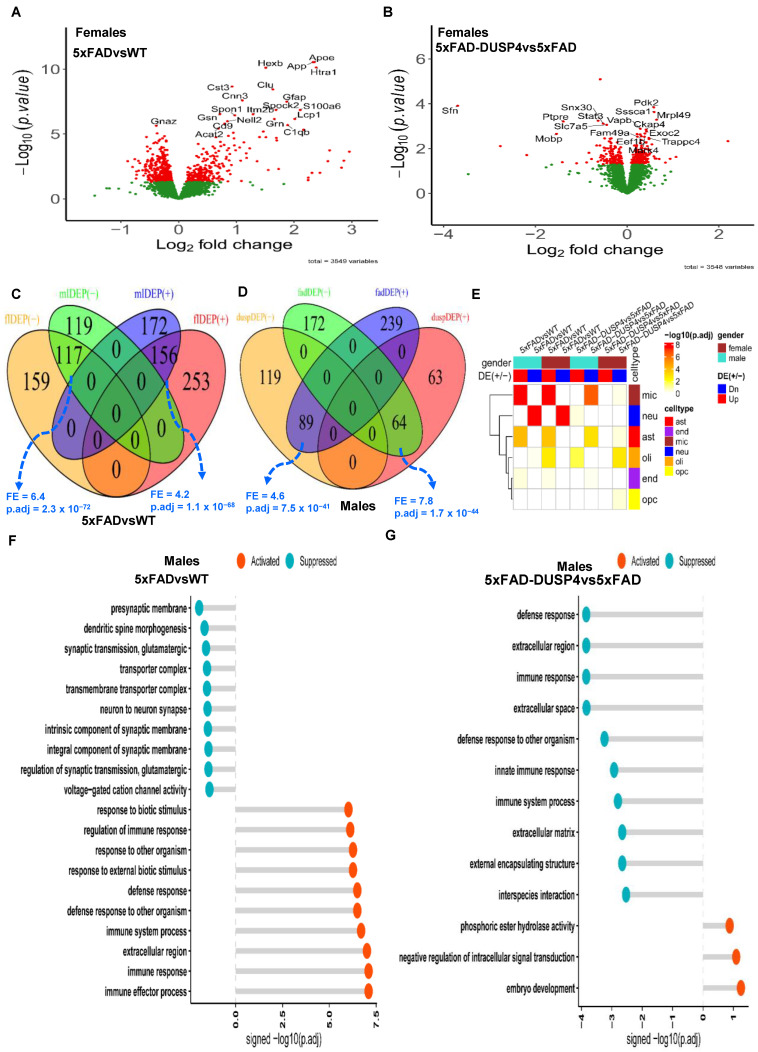
Analysis of differentially expressed proteins (DEPs). (**A**) Volcano plot showing the DEPs in 5xFADvsWT in female mice. (**B**) Volcano plot showing the DEPs in 5xFAD-DUSP4vs5xFAD female mice. In (**A**,**B**), each dot represents a protein, and highlighted are the top-ranked 10 DEPs in each comparison. Dots in red are DEPs, whereas dots in green are not differentially expressed proteins. (**C**) Venn diagram showing the overlap of the DEPs in 5xFADvsWT between male and female mice. mlDEP(-) and mlDEP(+) are down- and up-regulated DEPs in males. flDEP() and flDEP(+) are down- and up-regulated DEPs in females. (**D**) Venn diagram showing the overlapping of DEPs between 5xFADvsWT and 5xFAD-DUSP4vs5xFAD in male mice. fadDEP() and fadDEP(+) are down- and up-regulated DEPs in 5XFAD. duspDEP() and duspDEP(+) are down- and up-regulated DEPs in DUSP4 overexpression. FE, fold enrichment for the intersection between two DEPTM signature sets. p.adj, BH-adjusted *p*-value showing the statistical significance of the enrichment. (**E**) Enrichment of various mouse DEP lists from the present study in the published reference mouse cell-type signatures. The reference mouse cell-type signatures were curated and described in [50], which include six gene signatures that are specifically expressed in microglia (mic), neuron (neu), astrocytes (ast), oligodendrocytes (oli), endothelial cells (end), and oligodendrocyte precursor cells (opc), respectively. (**F**,**G**) GO enrichment analysis on DEPs of 5xFADvsWT (**G**) and 5xFAD-DUSP4vs5xFAD (**F**) in male mice. X-axis, −log10(p.adj) split by enrichment groups, activated (positive) vs. suppressed (negative). Y-axis, GO (gene ontology) terms. The higher of a −log10(p.adj) value the more likely the DEP signature is associated with a GO pathway.

Together, we quantified 4459 distinct proteins over the 46 samples. After QC (see Section 2), we obtained 3578 unique proteins. We performed DEP analysis to reveal the mouse proteome impacted by the 5xFAD transgene and DUSP4 overexpression. We identified 685 and 564 DEPs comparing 5xFADvsWT for female and male mice, respectively (Figure 2A; Supplementary Figure S1A; Supplementary Data S2A,B). We detected more DEPs that were down-regulated than up-regulated in 5xFADvsWT for mice of both sexes (Supplementary Figure S2). As expected, the expression of APP was substantially elevated in 5xFAD mice of each sex (Figure 2A; Supplementary Figure S1A; Supplementary Data S2A,B). In comparing 5xFAD-DUSP4vs5xFAD, we found 295 and 335 DEPs for female and male mice, respectively (Figure 2B; Supplementary Figure S1B; Supplementary Data S2C,D). In contrast to the comparison of 5xFADvsWT, we detected more up-regulated DEPs than down-regulated ones in 5xFAD-DUSP4vs5xFAD in each sex (Supplementary Figure S2). As anticipated, DUSP4 protein levels were markedly increased in 5xFAD-DUSP4vs5xFAD for both female (fold-change (FC) = 6.7, *p* = 0.05) and male (FC = 22.8, *p* = 4.7 × 10^−6^) mice, respectively (Supplementary Data S2C,D). Note that the APP protein expression was not altered in 5xFAD-DUSP4vs5xFAD.

We compared the DEP signatures across different comparisons for each sex. We separated up-regulated proteins from down-regulated ones to examine consistency in the directionality of protein expression changes. For each comparison, we observed significant overlap between the male and female DEP signatures in the direction of protein expression changes and insignificant overlap in the opposite directions (Figure 2C; Supplementary Figure S3A). For example, the up-regulated signatures of males and females in 5xFADvsWT significantly overlap (fold enrichment (FE) = 4.2, FDR = 1.1 × 10^−68^; Figure 2C) and the down-regulated signatures of males and females in 5xFAD-DUSP4vs5xFAD also significantly overlapped (FE = 1.8, FDR = 0.02; Supplementary Figure S3A). In contrast, in male mice, the up-regulated signature in 5xFAD-DUSP4vs5xFAD significantly overlaps the down-regulated signature in 5xFADvsWT (FE = 7.8, FDR = 1.7 × 10^−44^; Figure 2D). Similar results were observed in female mice (Supplementary Figures S2 and S3B). These results show that DUSP4 overexpression reverses the abnormal proteomic changes in the 5xFAD mice in comparison with the wildtype mice: in male mice, ~27% of the DEPs were reversed, whereas ~15% of the DEPs in female mice were reversed upon the DUSP overexpression (Supplementary Figure S2).

We further looked into the DEPs for cell-type specificity. We observed that the down-regulated signatures were enriched for the markers of neurons. In contrast, the up-regulated signatures were most enriched for the markers of microglia and astrocytes in 5xFADvsWT in both sex groups (Figure 2E), consistent with some previous findings of up-regulated immune response and neuronal damage, and down-regulated synaptic transmission [49]. However, in 5xFAD-DUSP4vs5xFAD, we found that the down-regulated signatures were enriched for the markers of microglia and astrocytes in both sex groups, whereas the up-regulated signature in only males was enriched for the neuronal markers (Figure 2E). Thus, overexpression of DUSP4 affected all the major brain cell types, albeit with differences in enrichment significance across sex groups (Figure 2E). 

We also examined biological pathways and functional processes in which these DEPs participated. In male 5xFAD mice, the immune and defense response was activated while neuronal and synaptic functions were suppressed (Figure 2F). Similar results were observed for female 5xFAD mice (Supplementary Figure S4A). We then examined the effect of DUSP4 overexpression in 5xFAD mice. In male mice, DUSP4 overexpression activated pathways like intracellular signal transduction while it suppressed immune and defense responses that were activated in 5xFAD mice (Figure 2G). However, in females, DUSP4 overexpression affected a different set of pathways (Supplementary Figure S4B). Note that many pathways suppressed via DUSP4 overexpression in female mice (e.g., apoptotic process) are detrimental to cell functions (Supplementary Figure S4B). These results revealed sex-specific functions of DUSP4.

### 3.2. DUSP4 Overexpression Caused Significant Changes in Differentially Expressed Post-translational Modification (DEPTM) Sites

We preprocessed the mass spectrometry (MS)-based phosphoproteome profiling using the R package PhosPiR, which removed MaxQuant-marked reverse sequences and potential contaminants, and have summarized the intensities for each phosphosite entry, termed PTM site (see Section 2). The expression level (intensity) at each PTM site was obtained from quantile normalization and low-rank approximation imputation [32]. We removed any PTM site with no gene name or PTM position information. The expression was further log2-transformed for the downstream analysis. 

**Figure 3:**
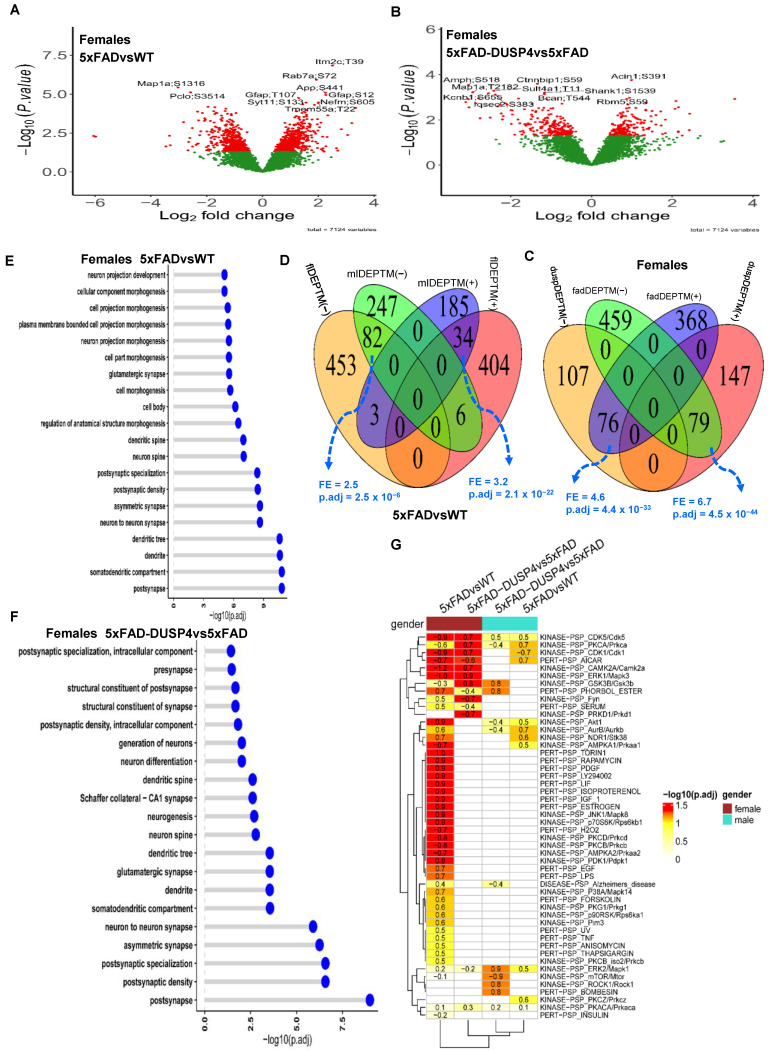
Analysis of differentially expressed PTM (DEPTM). (**A**,**B**) Volcano plots visualizing DEPTMs in 5xFADvsWT (**A**) and 5xFAD-DUSP4vs5xFAD (**B**) in female mice, respectively. In (**A**) and (**B**), each dot represents a protein and highlighted are top-ranked DEPTMs. Dots in red are DEPTMs that are significantly different expressions in the comparisons, whereas dots in green are not differentially expressed PTMs. Highlighted are the top 10 most significant DEPTMs. (**C**) Venn diagram showing the overlapping of DEPTMs between 5xFADvsWT and 5xFAD-DUSP4vs5xFAD in female mice. fadDEPTM(-) and fadDEPTM(+) are down- and up-regulated DEPTMs in 5XFAD. duspDEPTM(-) and duspDEPTM(+) are down- and up-regulated DEPTMs in DUSP4 overexpression. (**D**) Venn diagram showing the overlapping of DEPTMs in 5xFADvsWT between male and female mice. mlDEPTM(-) and mlDEPTM(+) are down- and up-regulated DEPTMs in males. flDEPTM(-) and flDEPTM(+) are down- and up-regulated DEPTMs in females. FE, fold enrichment for the intersection between two DEPTM signature sets. p.adj, BH-adjusted *p*-value showing the statistical significance of the enrichment. (**E**,**F**) GO enrichment analysis on the DEPTMs of 5xFADvsWT (**E**) and 5xFAD-DUSP4vs5xFAD (**F**) in female mice. Y-axis, GO terms. X-axis, −log10(p.adj), the higher of which, the more likely the DEPTM signature is associated with the GO pathways. (**G**) PTM site enrichment analysis on various DEPTM signatures. Highlighted numbers are the score of fold enrichment. The color bar showing the −log10(p.adj) for the enrichment score: The higher the value, the more likely the DEPTM signature is relevant to the PTM site signature in the database.

We obtained 7124 distinct PTMs across the 46 samples, spanned 2222 unique proteins, averaging about 3 PTM sites per protein. We performed differential expression analysis on all the PTMs. We identified 982 and 557 DEPTMs in 5xFADvsWT for female and male mice, respectively (Figure 3A; Supplementary Figure S5A; Supplementary Data S3A,B). We detected more DEPTMs that were up-regulated than down-regulated in 5xFADvsWT in both sex groups (Supplementary Figure S6). In comparing 5xFAD-DUSP4vs5xFAD, we found 409 and 425 DEPTMs for female and male mice, respectively (Figure 3B; Supplementary Figure S5B; Supplementary Data S3C,D). In contrast to the comparison of 5xFADvsWT, we detected more down-regulated than up-regulated DEPTMs in the 5xFAD-DUSP4vs5xFAD for mice of either sex (Supplementary Figure S6). We then compared the DEPTM signatures across different comparisons in each sex in the same way we conducted the DEP analysis (see above). Overall, a similar trend was observed for the DEPTMs as for the DEPs (Figure 3C,D; Supplementary Figure S7). In each comparison (5xFADvsWT or 5xFAD-DUSP4vs5xFAD), female and male mice shared a significant portion of DEPTMs with the same directionality, whereas in each sex, 5xFADvsWT and 5xFAD-DUSP4vs5xFAD showed significant overlap between their DEPTMs but with opposite directionality (Figure 3C,D; Supplementary Figure S7). We further quantified the proportion of DEPTMs that are reversed in expression via DUSP4 overexpression. We found that female mice had a slightly higher percentage of DEPTMs reversed (15%) compared to males (14%; see Supplementary Figure S6). These results again suggested that DUSP4 overexpression might reverse the effects of the 5xFAD transgene on mice at the phosphoproteome level.

We further explored the pathways in which the DEPTMs were involved. Since proteins may possess multiple phosphorylation sites, we collapsed the DEPTM sites onto their respective protein levels. We define a differentially phosphorylated protein (DPP) as containing at least one DEPTM. We obtained 665 and 418 DPPs in 5xFADvsWT for female and male mice, respectively, and 327 and 340 DPPs in 5xFAD-DUSP4vs5xFAD for female and male mice, respectively. As shown in Figure 3E,F, the most affected pathways are involved in neuronal processes and synaptic function for the DPPs (DEPTMs) across the comparisons in each sex (Supplementary Figure S8A,B), suggesting that both 5xFAD and DUSP4 might often influence the phosphorylation state of the proteins that are relevant to neuronal and synaptic function. 

To delve more deeply into the signals represented in our phosphoproteome profiling, we applied the site-centric pathway analysis [33] on our PTMs via the algorithm as described in the R package GSVA [34] (see Section 2). We examined how the PTMs are enriched for the PTM site-specific phosphorylation signatures [33] (PTMsigDB). As shown in Figure 3G, in female 5xFADvsWT, the PTMs were enriched over more than half of the PTM sets in the mouse PTMsigDB. The top-ranked kinase PTM sets are KINASE-PSP_CAMK2A/Camk2a, KINASE-PSP_ERK1/Mapk3, and KINASE-PSP_JNK1/Mapk8 [51] (Figure 3G), which are critical in AD neuropathogenesis. Strikingly, the PTMs from 5xFAD-DUSP4vs5xFAD in female mice are enriched for the PTM sets in the mouse PTMsigDB yet with an opposite directionality in enrichment score (ES) (Figure 3G), highlighting that DUSP4 overexpression in 5xFAD mice normalized the dysregulation of the PTM sets in 5xFAD mice in comparison with WT mice. In contrast, in male mice, the enrichment of PTMs in the mouse PTMsigDB was not very evident in spite of the enrichment in a few PTM sets (Figure 3G). These results further suggested that DUSP4 overexpression might counteract the effects of the 5xFAD transgene in mice in PTM site-centric pathways. 

### 3.3. DUSP4 Overexpression Resulted in Reduction in STAT3 in 5xFAD Mice

**Figure 4:**
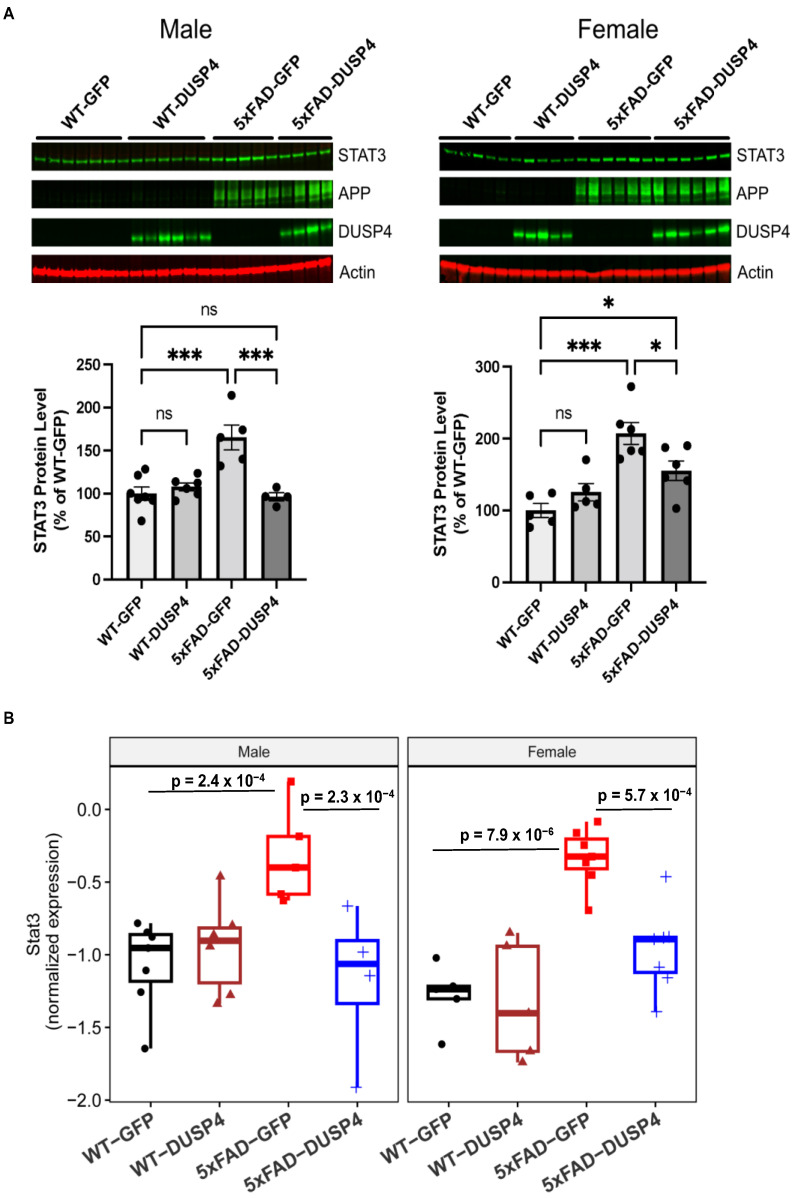
Western blot analyses of DEPs from wildtype (WT) and 5xFAD mice overexpressing GFP or DUSP4 to validate proteomic results. (**A**) Western blot analyses of hippocampal STAT3, human APP (hAPP), and DUSP4 protein levels in male WT and 5xFAD mice overexpressing GFP or DUSP4 (Left panel). *n* = 4–7 mice per group. Western blot analyses of hippocampal STAT3, hAPP, and DUSP4 protein levels in female WT and 5xFAD mice overexpressing GFP or DUSP4 (Right panel). *n* = 5–6 mice per group. Error bars represent means ± SEM. Statistical analyses were performed using a one-way ANOVA followed by a Tukey’s post hoc test, * *p* < 0.05, *** *p* < 0.001; ns = insignificant. (**B**) Boxplots showing the normalized expression of STAT3 in proteome profiling. Left and right are for Stat3 expression in male and female WT and 5xFAD mice overexpressing the mock GFP or DUSP4, respectively. Statistic *p*-values were shown for the comparisons that have a significant difference in Stat3 protein expression. The numbers of mice are the same as used in Figure 4A,B. Original figures can be found in supplementary materials.

Hippocampal STAT3, human APP (hAPP), and DUSP4 protein levels were significantly altered in our proteomics data. Interestingly, STAT3 has been implicated in AD-associated neuroinflammation, and it has been reported that inhibition of STAT3 ameliorates AD-associated neuroinflammation [52]. In addition, we show that Stat3 is one of the most downregulated DEPTMs and DEGs following DUSP4 overexpression in 5xFAD, utilizing both phosphoproteomics analyses in the current study and transcriptomics analyses in our published study [22]. Therefore, STAT3 could be a potential mediator by which DUSP4 regulates AD-associated neuroinflammation. We then further validate the changes in these proteins using western blot analyses. The results showed that hippocampal STAT3 protein levels were increased by about 110% in female 5xFAD mice overexpressing GFP (5xFAD-GFP), while male 5xFAD-GFP increased by about 65%, compared to age- and sex-matched wildtype mice overexpressing GFP (WT-GFP) (Figure 4A). STAT3 protein levels were reduced by about 65% in both female and male 5xFAD overexpressing DUSP4 (5xFAD-DUSP4) compared to age- and sex-matched 5xFAD-GFP (Figure 4A). Although STAT3 protein levels were significantly reduced in female 5xFAD-DUSP4 compared to female 5xFAD-GFP, levels were significantly higher than female WT-GFP, while Stat3 protein levels in male 5xFAD-DUSP4 showed no significant differences compared to male WT-GFP (Figure 4A). Strikingly, these results nearly replicated those we obtained from the proteomic analysis (Figure 4B) regarding Stat3 expression in the different mouse genotypes (Figure 4B): Stat3 levels were dramatically increased in 5xFAD-GFP compared to WT-GFP, which was restored by DUSP4 overexpression in 5xFAD (Figure 4B). Furthermore, our western blot analyses confirmed the DUSP4 overexpression in both female and male mice administered with AAV-DUSP4. In addition, western blot analyses detected the hAPP protein only in 5xFAD transgenic mice, which confirmed 5xFAD genotypes. Together, these results validate the proteomics analyses. 

### 3.4. The DUSP4 DEP and DEPTM Signatures Are Enriched in Human AD Protein Networks

**Figure 5:**
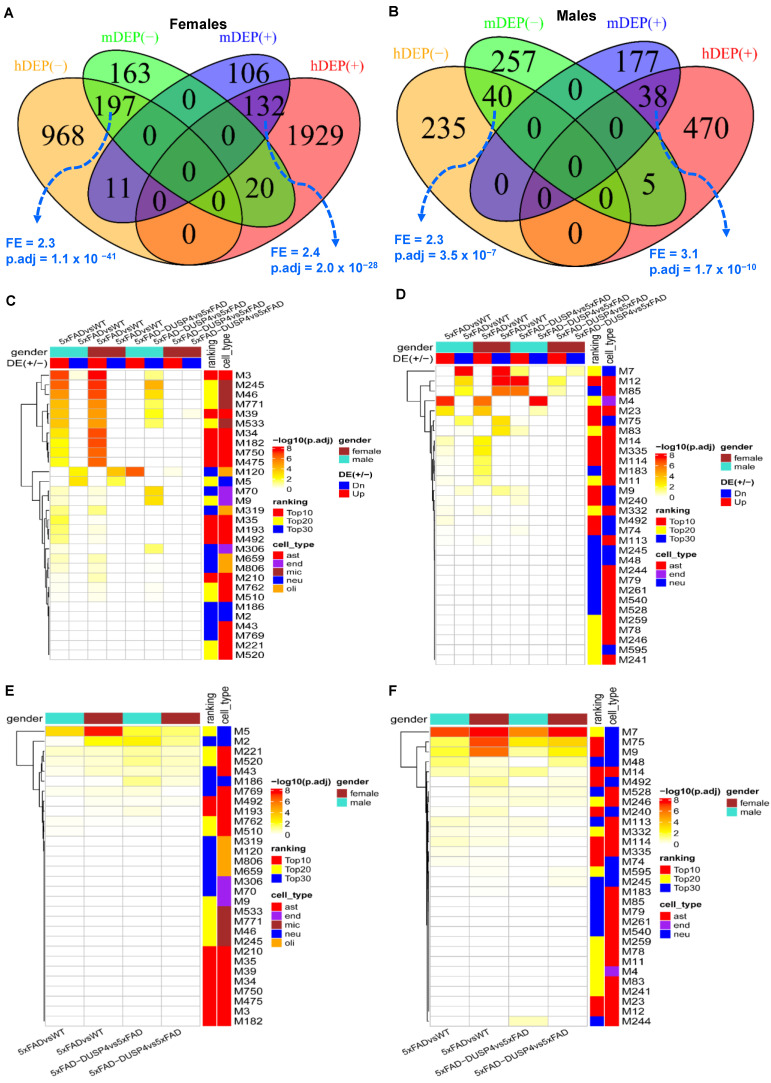
Integration of the DEPs and DEPTMs in mice with the human co-expression networks. (**A**) Venn diagram revealing the overlap between female mouse DEPs in 5xFADvsWT and human female-specific DEPs in AD vs. NL (The DEPs identified in human females). (**B**) Venn diagram showing the overlap between male mice DEPs in 5xFADvsWT and human male-specific DEPs in AD vs. NL (the DEPs identified in human males). In (**A**,**B**), mDEP(+) and mDEP(−) denote up- and down-regulated DEPs in mice, respectively, whereas hDEP(+) and hDEP(−) denote up- and down-regulated DEPs in humans, respectively. (**C**,**D**) Heatmaps highlighting the enrichment of the mouse DEP signatures over human proteomics MEGENA co-expression networks for the MSBB (**C**) and ROSMAP (**D**) cohorts, respectively. (**E**,**F**) Heatmaps highlighting the enrichment of the mouse DEPTMs over human proteomics MEGENA co-expression networks for the MSBB (**E**) and ROSMAP (**F**) cohorts, respectively. DE(+/−)denotes up- or down-regulated DEPs, respectively. Ranking denotes the categories of the module ranking order in relevance to AD: Top10, Top20, and Top30 represent top-ranked 10, 20, and 30 AD modules, respectively. Cell type is the cell type that is the most enriched in each module. ast, astrocytes; neu, neurons; endo, endothelial cells; mic, microglia; olig, oligodendrocytes. Because a protein may have more than one DEPTM site, we collapsed the DEPTMs to their respective protein levels; that is, a protein represents all the DEPTMs that belong to it. Each field in the heatmap represents the intersection between a DEP or DEPTM signature over a module (clusters of genes) in the network. Only the top 30 AD associated modules are shown.

We first compared the mouse DEP signatures in the present study with the human DEPs in AD that were derived from the proteomics profiling in the parahippocampal gyrus (PHG) of the MSBB cohort [38,41]. We stratified the human subjects over sex and thus obtained the sex-specific DEPs in AD vs. normal healthy individuals (NL) (Supplementary Data S4, and Section 2). The mouse DEPs in 5xFADvsWT significantly overlapped the human DEP signatures, with the same directionality in both sexes, though the overlap of male signatures was much less significant (Figure 5A,B). On the other hand, the DEP signatures in 5xFAD-DUSP4vs5xFAD in the male mice have marginally significant overlap with the human male DEP signatures in the opposite directions (Supplementary Figure S9A), while the signatures from the female mice do not significantly overlap the respective human signatures (Supplementary Figure S9B). These results validated the mouse DEPs we identified and suggested that our findings from the mouse proteomics might be relevant to human AD neuropathology. 

We projected the mouse DEP signatures onto the MEGENA co-expression networks from the human proteomics [41] to further understand their functional relevance to human AD. In the MSBB protein co-expression network, more than half (>15) of the top 30 AD-associated modules were enriched for the mouse DEPs from 5xFADvsWT of both sexes (Figure 5C). The up-regulated DEPs in both male and female mice are enriched in the astrocyte (M3) and microglia modules (M245), while the down-regulated DEPs overlap significantly with the neuronal modules (M5) (Figure 5C). We also observed the enrichment of the DEPs from 5xFAD-DUSP4vs5xFAD in the network, especially the down-regulated DEPs in the male mice (Figure 5C). Similar results were found in the ROSMAP MEGENA network (Figure 5D). These results further validated the relevance of the mouse DEPs to human AD and were consistent with the aforementioned cell-type enrichment analysis (Figure 2E). 

Furthermore, the mouse DEPTMs are also enriched in a number of top-ranked AD modules in the MSBB (Figure 5E) and ROSMAP (Figure 5F) protein co-expression networks. Importantly, the most enriched modules are neuron-specific (M5 and M2 in the MSBB cohort, Figure 5E; M7 and M75 in the ROSMAP cohort, Figure 5F). These results are consistent with the previous pathway enrichment analysis (Figure 3E,F) and indicate that the DEPTMs are often involved in neuronal and synaptic functions.

### 3.5. DUSP4 Protein-Centered Networks Are Sex-Specific

**Figure 6:**
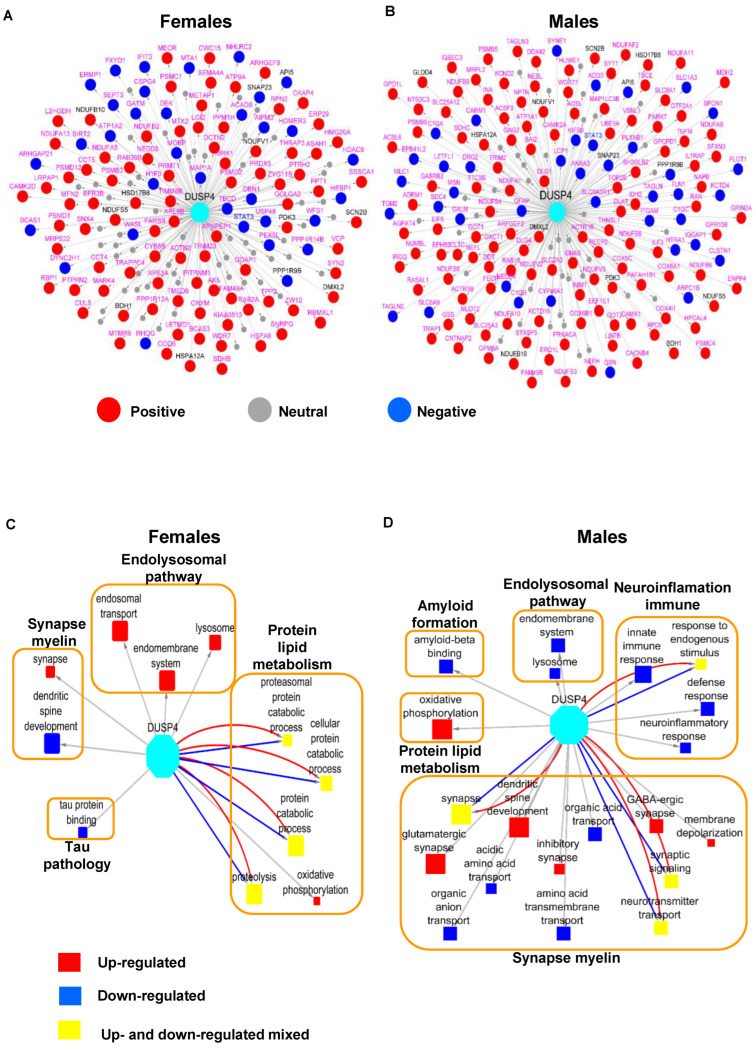
The DUSP4 protein-centric network analysis. The networks were inferred using the DEP signatures from 5xFAD-DUSP4vs5xFAD for female (**A**) and male (**B**) mice. Each node represents a gene (protein). Labeled in pink are the genes that are specifically associated with DUSP4 in females (**A**) and males (**B**), respectively, whereas those in grey are common to both sexes. Red and blue nodes are positively- and negatively-associated with DUSP4, respectively, whereas grey nodes are not associated with, thus, not impacted by DUSP4. (**C**,**D**) DUSP4 signaling maps in female (**C**) and male (**D**), respectively, are shown. Each filled box denotes a GO term whose size is proportional to its enrichment for DUSP4-centric signatures. Red and blue nodes indicate the pathways enriched for solely positive and negative DUSP4-centric signatures, respectively, whereas yellow ones are pathways enriched for both positive and negative DUSP4-centric signatures. The large unfilled boxes denote the parent categories of GO terms in AD. The DUSP4-centric networks reveal the proteins/genes that are regulated by or associated with DUSP4, and the DUSP4 signal maps further show the biological processes the genes in the DUSP4-centric network are involved in.

To formally identify the genes that are co-regulated with DUSP4 in AD, we leveraged a number of human AD cohorts as previously described [43] by examining the genes with significant correlations with DUSP4. We intersected the mouse DEP signatures in 5xFAD-DUSP4vs5xFAD with the human DUSP4-associated genes and further constructed DUSP4 protein-centric networks for each sex (Figure 6A,B). There are more proteins positively correlated with the DUSP4 protein/gene than those negatively correlated with the DUSP4 protein/gene (Figure 6A,B). Impressively, the majority of the DUSP4-associated proteins are specifically expressed in either females (Figure 6A) or males (Figure 6B). Based on the sex-specific DUSP4-centric networks, we constructed the sex-specific DUSP4 signal maps (Figure 6C,D). As shown in Figure 6C, in females, DUSP4 is often involved in protein and lipid metabolism, in contrast to its involvement in synapse and myelin functions in males (Figure 6D). DUSP4 participates in endolysosomal pathways in both male and female mice but in the opposite directions (Figure 6C,D). In summary, the results demonstrate that DUSP4 plays important roles in AD pathogenesis by regulating biological processes and functions shared by two sexes or distinct in each sex. 

## 4. Discussion

In the present study, we investigated the proteins and phosphorylation sites that are modulated in 5xFAD mice and examined the sex-specific impacts of DUSP4 overexpression on the 5xFAD proteome/phosphoproteome. In 5xFAD mice, a substantial number of proteins were up- or down-regulated in both male and female mice, and they were involved in AD-related biological processes, such as activated immune response (up-regulated in microglia and astrocytes) or suppressed synaptic activities (down-regulated in neurons). Upon DUSP4 overexpression, those dysregulated proteins and pathways (for example, immune response and defense response) were rescued. For the phosphoproteome, we detected an array of phosphorylation sites that are associated with 5xFAD and DUSP4 overexpression in each sex. However, the 5xFAD- and DUSP4-associated phosphorylation changes were in the opposite directions. Strikingly, both 5xFAD- and DUSP4-associated phosphorylation changes occurred mainly in neurons, which were predicted to regulate neuronal processes and synaptic function. Site-centric pathway analysis revealed that both the 5xFAD- and DUSP4-phosphorylation sites were enriched for a number of kinases in females but only a limited number of kinases in male mice. Our study represents, to our knowledge, the first examination of the proteome and phosphoproteome that is modulated by DUSP4 and the determination of the significance of such modulation in AD. 

DUSP4 is a mitogen-activated protein kinase (MAPK) pathway regulator, which regulates a wide variety of cellular signaling pathways, including stress responses, differentiation, and apoptosis [53]. Intriguingly, transcriptomic profiling of hippocampal RNAs in patients with Alzheimer’s disease (AD) showed a downregulation of DUSP4 [54], suggesting a potential role for DUSP4 in AD-associated pathogenesis. Our previous study in the 5xFAD AD animal model indicated that hippocampal DUSP4 overexpression rescued spatial memory deficits in female 5xFAD mice but not in male 5xFAD mice [22]. In addition, transcriptomic profiling of 5xFAD mice overexpressing DUSP4 showed that differentially expressed genes (DEGs, false discovery rate (FDR) < 0.05), including Stat1, Stat2, and Ccl2 were downregulated in female 5xFAD-DUSP4 mice, while no DEGs (FDR < 0.05) were detected in male 5xFAD overexpressing DUSP4. Furthermore, enrichment analysis of DEGs predicted that neuroinflammatory, interferon, and extracellular signal-regulated kinase (ERK)/MAPK signaling pathways were regulated in female 5xFAD overexpressing DUSP4 [22]. While these transcriptomic data suggested a role for DUSP4 in AD-associated neuroinflammation, it is not clear how DUSP4 downregulated neuroinflammatory pathways. Consistent with our transcriptomic profiling of groups of mice with the same genotypes and AAV treatments, in the present study, we observed up-regulated STAT1 protein in 5xFAD, which was reported in human AD [55], whereas DUPS4 overexpression in 5xFAD female mice down-regulated STAT1 protein expression (Supplementary Data S2). Similar results were found in male mice, although the changes were not robust by comparison in terms of *p*-values. STAT2 and CCL2 were not profiled in the present study because of either low abundance at the protein expression level or large variation in expression among the samples, which caused their exclusion from further analysis. Overall, we observed significant overlaps between the 5xFAD- and DUSP4-associated signatures of both protein and phosphoprotein in female and male mice (Figure 2D and Figure 3C; Supplementary Figures S3B and S7A), but in the opposite directions, indicating that DUSP4 overexpression may reduce AD-related deficits by reversing the dysregulated genes/proteins in 5xFAD in comparison with WT in a sex-specific manner. Furthermore, there exist significant differences in the sex-specific DUSP4-centric networks and signal maps (Figure 6). Taken together, our results that demonstrate sex-specific differences in the response of the 5xFAD proteome and phosphoproteomce to DUSP4 overexpression further support previous observations that DUSP4 overexpression reduces amyloidopathy in both sexes but learning deficits only in female 5xFAD mice [22]. 

Microglia-associated neuroinflammation is characteristic of AD-associated pathology and was reported to be regulated by the ERK/MAPK signaling pathway [51]. Quantitative proteomics analyses showed that ERK1 and ERK2 were up-regulated in postmortem AD human brains, and phosphorylated ERK was also increased in isolated microglia from 5xFAD mice [51]. In addition, proteomics analyses of the hippocampus in 5xFAD mice have revealed several pro-inflammatory markers, including STAT3 [49], which can promote microglia-dependent neuroinflammation. For example, it was previously shown that deletion of microglial STAT3 in mice prevented microglia-dependent neuroinflammation [56]. The ERK/MAPK signaling pathway is a critical regulator of pro-inflammatory microglial activation, and microglial activation has been suggested as a contributor to the progression of AD [57]. In the present study, we found that DUSP4 overexpression in 5xFAD mice caused a reduction in STAT3 protein levels in both sexes (Figure 4 and Supplementary Data S2). We subsequently queried the protein and protein interaction (PPI) network [58] in AD and obtained a STAT3 subnetwork (Supplementary Figure S10). Impressively, the STAT3-subnetwork is enriched for a number of pathways critical to AD, such as amyloid formation, tau pathology, neuroinflammation, and synapse and myelin functions (Supplementary Figure S10). Thus, it is speculated that STAT3 is the connection point through which DUSP4 exerts its effects on AD, which might be one of the mechanisms underlying DUSP4 functionality that is shared in male and female mice. 

In the present study, we used the nominal *p* < 0.05 as the cut-off in order to maximize inclusion of proteins/phosphoproteins that are regulated by DUSP4 overexpression. First, we carefully followed the standard experimental protocols and data processing pipelines (see Section 2). As an example, we performed principal component analysis (PCA) on the protein and phosphoprotein expression data (Supplementary Figure S11), which was encouraging as it indicates that, in general, mouse samples can be grouped together concordant to their genotypes. Then, we inspected and validated some of the proteins that were known to be regulated by 5xFAD. For example, we observed the up-regulation of the APP [22,49], APOE [49], and STAT3 [49,52] proteins in the 5xFAD mice of either sex, which is not only consistent with previously reported studies but was further confirmed by our experimental validation (Figure 4) and the integration analysis with the human proteomics profiling (Figure 5). For DEPTMs, we observed that the phosphorylation site (APP;S441) in the APP protein was significantly up-regulated in both male and female 5xFAD mice (Figure 3A; Supplementary Data S3A,B). APP serine 441 has been inferred to be phosphorylated by a combination of experimental and computational evidence [59] (The mouse App entry P12023, UniProtKB at https://www.uniprot.org/). Strikingly, DUSP4 overexpression resulted in a decreased level of phosphorylation at this site (APP;S441) in 5xFAD mice of either sex (Supplementary Data 3C,D). S441 is found within the E2 dimerization domain of APP (aa374-565) (The mouse App entry P12023, UniProtKB at https://www.uniprot.org/). Whether S441 phosphorylation modulates antiparallel App dimer formation, heparin binding, and/or binding with other App interactors is to our knowledge unknown, although protein phosphorylation has been reported to modulate APP interactions [60] and to occur in the APP ectodomain [61]. Similarly, we also observed 26 PTMs in the tau protein (Mapt gene), some of which displayed significant association with 5xFAD or DUSP4 (Supplementary information; Supplementary Figure S12). As an additional line of evidence, in female 5xFAD mice, we observed high consistency between the DEPs from the present study and the DEGs from our previous work [22] (Supplementary Figure S13, Supplementary results and discussion). These results and evidence together support this cut-off (nominal *p* < 0.05) as an effective criterion in determining the protein/phosphoprotein signatures regulated by DUSP4, albeit we cannot rule out any exceptions due to false discovery. 

In the present study, we investigated the sex-specific impact of DUSP4 overexpression on proteome and phosphoproteome in 5xFAD mice. To determine whether such sex-specific molecular responses to DUSP4 overexpression were due to the sex difference in DUSP4 protein expression, we performed differential protein expression analysis of DUSP4 in male vs. female mice with overexpression of DUSP4. However, there was no significant difference in DUSP4 expression between male and female mice (*p* = 0.59, Supplementary Figure S15). Therefore, our data does not support the hypothesis that our observed sex-specific molecular changes were due to the differential expression of DUSP4 between male and female mice. 

## 5. Conclusions

Our study thoroughly investigated and characterized the DUSP4-associated proteome and phosphoproteome, revealing the shared and sex-specific molecular mechanisms through which DUSP4 functions in an AD mouse model. 

## Data Availability

The mouse proteomics and phosphoproteomics profiling data are available via syn52138250 at the AD Knowledge Portal (https://adknowledgeportal.synapse.org). The raw data are available through syn31952297 and syn31947506 at the AD Knowledge Portal. The AD Knowledge Portal is a platform for accessing data, analyses, and tools generated by the Accelerating Medicines Partnership Alzheimer’s Disease (AMP-AD) Target Discovery Program and other NIA-supported programs to enable open science practices and accelerate translational learning. The data, analyses, and tools are shared early in the research cycle without a publication embargo on secondary use. Data are available for general research use according to the following requirements for data access and data attribution (https://adknowledgeportal.synapse.org/DataAccess/Instructions, accessed on 17 November 2023). All the analytic scripts are available upon request.

## Supplementary Materials

The following supporting information can be downloaded at: https://www.mdpi.com/article/10.3390/biom14010066/s1, Figure S1: Volcano plots for DEP analysis; Figure S2: Summary of the overlap and significance of the DEP signatures via the superExactTest; Figure S3: Venn diagrams highlighting the overlap of the DEP signatures; Figure S4: GO enrichment analysis on the DEP signatures; Figure S5: Volcano plots for DEPTM analysis; Figure S6: Summary of the overlap and significance of the DEPTM signatures via the superExactTest; Figure S7: Venn diagrams highlighting for the overlap of DEPTM signatures; Figure S8: GO enrichment analysis on the DEPTM signatures; Figure S9: Venn diagrams highlighting for the overlap of the DEP signatures between human and mouse; Figure S10: The DUSP4-STAT3 protein signal map; Figure S11: PCA analysis of the protein and phosphoprotein expression; Figure S12: Heatmap showing the significance and change in expression of PTMs in the tau protein (Mapt gene); Figure S13: Venn diagram highlighting for the overlap between DEP and DEG signatures for 5xFADvsWT; Figure S14: Venn diagrams highlighting for the overlap between DEP and DEG signatures for 5xFAD-DUSP4vs5xFAD; Figure S15: Boxplots showing DUSP4 protein expression in male vs. female mice in the proteomic profiling; Data S1: Mouse phenotypic data; Data S2A: Analysis of differentially expressed proteins (DEP) in 5xFADvsWT in female mouse; Data S2B: Analysis of differentially expressed proteins (DEP) in 5xFADvsWT male mouse; Data S2C: Analysis of differentially expressed proteins (DEP) in 5xFAD-DUSP4vs5xFAD in female mouse; Data S2D: Analysis of differentially expressed proteins (DEP) in 5xFAD-DUSP4vs5xFAD in male mouse; Data S3A: Analysis of differentially expressed PTM (DEPTM) in 5xFADvsWT in male mouse; Data S3B: Analysis of differentially expressed PTM (DEPTM) in 5xFADvsWT in female mouse; Data S3C: Analysis of differentially expressed PTM (DEPTM) in 5xFAD-DUSP4vs5xFAD in male mouse; Data S3D: Analysis of differentially expressed PTM (DEPTM) in 5xFAD-DUSP4vs5xFAD in female mouse; Data S4A: The male-specific DEP analysis in the human cohort MSBB; Data S4B: The female-specific DEP analysis in the human cohort MSBB.

## List of Abbreviations

DUSP4	dual specificity phosphatase 4
MAPK	mitogen-activated protein kinases
AD	Alzheimer’s disease
AAV	adeno-associated virus
WT	wildtype
MS	mass spectrometry
PTM	Post-translational modification
APOE	apolipoprotein E ε4
PBS	phosphate buffered saline
LFQ	label-free quantification
DEP	differentially expressed protein
DEPTM	differentially expressed post-translational modification
GSVA	Gene set variation analysis
GO	Gene ontology
MEGENA	Multiscale Embedded Gene Co-expression Network Analysis
FDR	false discovery rate
TMT	Tandem Mass Tag
