# Supplementary figures and images for: Proteomic Signaling of Dual-Specificity Phosphatase 4 (DUSP4) in Alzheimer’s Disease
Source: Biomolecules. 2024 Jan 3;14(1):66. doi: 10.3390/biom14010066 (PMC10813059; doi:10.3390/biom14010066)

# Male

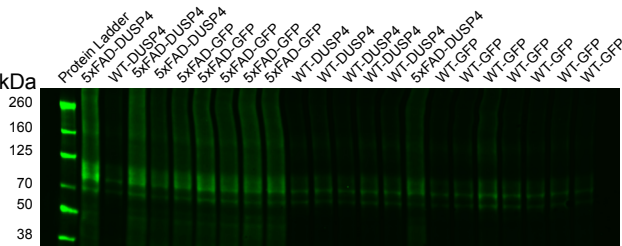

hAPP

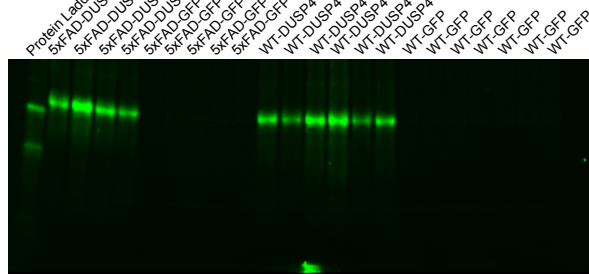

DUSP4

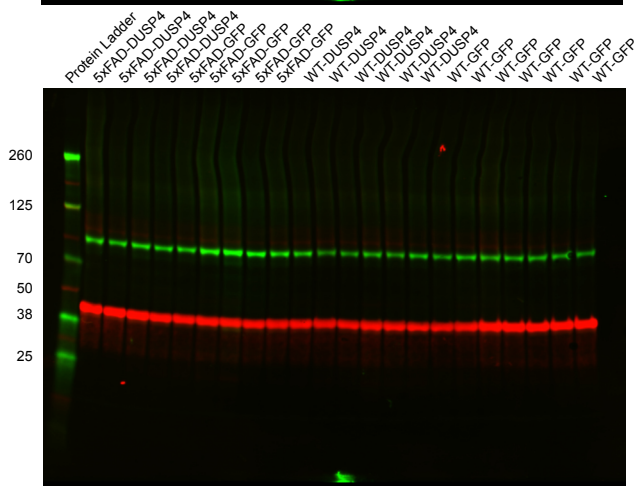

STAT3

Actin

# Female

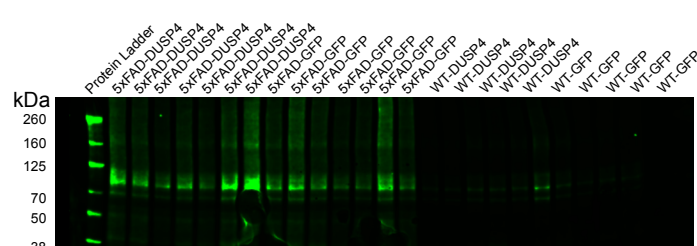

hAPP

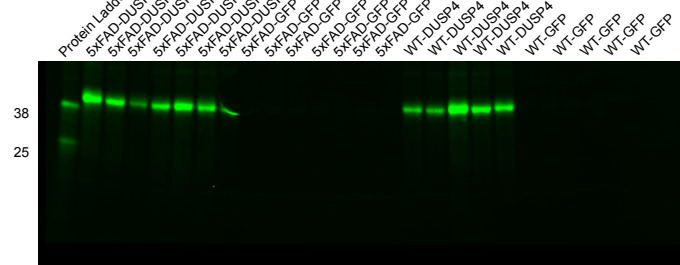

DUSP4

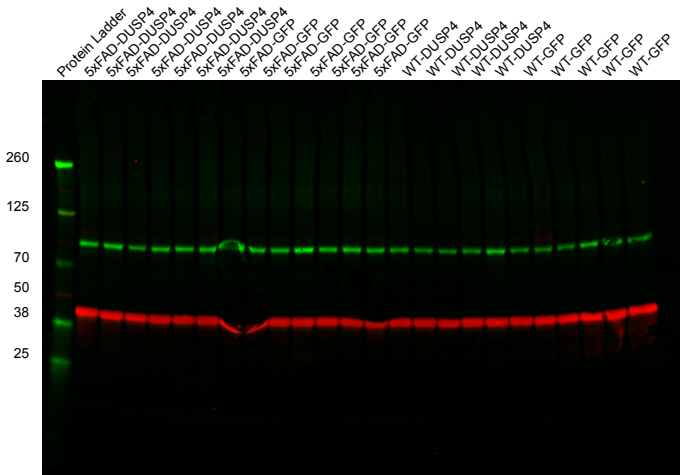

STAT3

Actin

Supplement: Supplementary file 1 [file biomolecules-14-00066-s001.zip › biomolecules-2751138-original-images.pdf]
